# Enhanced Osteogenic Differentiation of Human Primary Mesenchymal Stem and Progenitor Cultures on Graphene Oxide/Poly(methyl methacrylate) Composite Scaffolds

**DOI:** 10.3390/ma13132991

**Published:** 2020-07-05

**Authors:** Katarzyna Krukiewicz, David Putzer, Nicole Stuendl, Birgit Lohberger, Firas Awaja

**Affiliations:** 1 Department of Physical Chemistry and Technology of Polymers, Silesian University of Technology, 44-100 Gliwice, Poland; 2 Experimental Orthopedics, Department of Orthopedic Surgery, Medical University Innsbruck, 6020 Innsbruck, Austria; david.putzer@i-med.ac.at; 3 Department of Orthopedics and Trauma, Medical University of Graz, 8036 Graz, Austria; nicole.stuendl@medunigraz.at (N.S.); birgit.lohberger@medunigraz.at (B.L.); 4 School of Medicine, National University of Ireland, H91 CF50 Galway, Ireland; 5 Engmat Ltd., Clybaun Road, H91 P96H Galway, Ireland

**Keywords:** bone regeneration, graphene oxide, mesenchymal stem and progenitor cells, osteogenic differentiation, poly(methyl methacrylate)

## Abstract

Due to its versatility, small size, large surface area, and ability to interact with biological cells and tissues, graphene oxide (GO) is an excellent filler for various polymeric composites and is frequently used to expand their functionality. Even though the major advantage of the incorporation of GO is the enhancement of mechanical properties of the composite material, GO is also known to improve bioactivity during biomineralization and promote osteoblast adhesion. In this study, we described the fabrication of a composite bone cement made of GO and poly(methyl methacrylate) (PMMA), and we investigated its potential to enhance osteogenic differentiation of human primary mesenchymal stem and progenitor cells. Through the analysis of three differentiation markers, namely alkaline phosphatase, secreted protein acidic and rich in cysteine, and bone morphogenetic protein-2 in the presence and in the absence of an osteogenic differentiation medium, we were able to indicate a composite produced manually with a thick GO paper as the most effective among all investigated samples. This effect was related to its developed surface, possessing a significant number of voids and pores. In this way, GO/PMMA composites were shown as promising materials for the applications in bone tissue engineering.

## 1. Introduction

Since its discovery in 2004 [1], graphene has drawn immense attention of the scientific community and has become an object of intensive research. Due to its high planar surface area, superior mechanical strength, outstanding optical properties, as well as remarkable thermal and electrical conductivity [2,3,4], graphene has been widely used in a variety of applications, including transparent conductors, ultrafast transistors, precise biosensors, and tissue scaffolds [5]. The potential of graphene has been further expanded by introducing to its structure a variety of functional group, resulting in the fabrication of graphene oxide (GO). GO is usually produced by the oxidation of graphite, resulting in a partial breaking of sp^2^ bonds present in its structure and subsequent increase in the distance between carbon layers [3]. Therefore, GO possesses both hydrophobic (due to the presence of pristine graphite structure) and hydrophilic (due to the presence of hydroxyl, epoxy, carbonyl, and carboxyl groups) parts, and is characterized by affinity for aromatic rings, excellent aqueous processability, amphiphilicity, ease of functionalization, and biocompatibility [3,5]. Consequently, GO has been marked as an excellent material for numerous biomedical applications, including the design of biosensors [5], drug delivery systems [6], antimicrobial coatings [7], cell imaging platforms [8], and in gene therapy [9].

Due to its versatility, small size, large surface area, and ability to interact with biological cells and tissues, GO is an excellent filler for various polymers and is frequently used to expand their functionality. For instance, Wan et al. [10] reported an increase in the tensile strength, modulus, and energy at break, as well as the improvement in bioactivity during biomineralization simultaneously with maintaining high porosity when an electrospun poly(ε-caprolactone) membrane was reinforced with GO nanoplatelets. Also Baradaran et al. [11] observed the increase in elastic modulus and fracture toughness, as well as the promotion in osteoblast adhesion and proliferation when GO was used as a filler for hydroxyapatite nanosheets. On the other hand, calcium phosphate mineralized graphene oxide/chitosan scaffolds were found to express biomimicry, providing a suitable environment for cell adhesion and growth, and maintaining high mechanical strength [12]. GO was also demonstrated to act as an excellent filler for such polymer matrices as poly(vinyl alcohol) [13], poly(carbonate urethane) [14], hyaluronic acid [15], and poly(acrylic acid) [16], resulting in the formation of robust composite materials with applicability in biomedical engineering.

Poly(methyl methacrylate) (PMMA) is a non-toxic thermoplastic polymer possessing a very good toxicological safety record in biomedicine [17]. PMMA is frequently used as a screw fixation in bone, bone cement, filler for bone cavities and skull defects, as well as vertebrae stabilization in osteoporotic patients [18]. The exceptional applicability of PMMA in orthopedic and dental applications is caused by its good processability, handling properties, biocompatibility, suitable mechanical strength, and Young’s modulus [19]. Despite its numerous merits, the common complications of using PMMA is bone resorption observed after implantation, which is the effect of the formation of a weak-link zone derived from not sufficient interactions between cement and a bone [20]. Therefore, the modification of PMMA has become a very active area of research. A promising way to improve the biological performance of PMMA is to blend it with an antibiotic, e.g., gentamycin [21]. This modification approach is nowadays a well established strategy that allows prevention of periprosthetic infections and osteomyelitis. Another way to enhance the performance of PMMA is to incorporate in its structure a filler with a particular functionality. For instance, loading of PMMA with multiwalled carbon nanotubes could significantly improve the mechanical properties and reduce the exothermic polymerization reaction of the bone cement [22]. The use of tri-calcium phosphate and chitosan as inorganic/organic additives to PMMA decreased polymer curing temperature, extended setting time, and increased weight loss and porosity after degradation and, among all, promoted better osteointegration than pure PMMA [23]. Also graphene and graphene oxide have been used as fillers to PMMA [24], improving the mechanical properties of PMMA, particularly its fracture toughness and fatigue performance. What was interesting, GO was found to outperform graphene and provide greater enhancements due to its high functionalization that increased the interfacial adhesion between a filler and PMMA matrix. Simultaneously, the presence of graphene or GO was not found to have any negative effect on the biocompatibility of PMMA composites, potentially allowing their further clinical progression [25].

In this paper, the potential of GO/PMMA composites for the application in bone tissue engineering is assessed by the analysis of three differentiation markers expressed by human primary mesenchymal stem and progenitor cells (hMSPCs) cultured on the top of the composites. By performing the cell culture in the presence and absence of a specific induction medium, we were able to determine the efficiency of osteogenic differentiation of hMSPCs cultured on four types of GO/PMMA scaffolds, differing in the thickness of GO paper as well as the method of fabrication of the composites. Microscopic analysis of the surface of materials allowed investigation of the biological behavior of the materials with respect to their surface morphology. 

## 2. Materials and Methods 

### 2.1. Fabrication of GO/PMMA Composites

For the production of graphene oxide (GO) paper, 4 mg/mL suspension of GO flakes (GO monolayer content > 95%, oxygen content between 40% and 50%) in water was purchased from Graphenea (San Sebastián, Spain). GO suspension was inserted into Petri dishes with serological pipettes. The GO dispersion was dried in a shaking incubator with air fan for 48 h and inserted into an oven at 180 °C for 20 min. By changing the volume of GO suspension, two types of GO paper were fabricated, i.e., thin (8 mL of GO suspension) and thick (10 mL of GO suspension), designated as GO_(A)_ paper and GO_(B)_ paper, respectively.

**Scheme 1:**
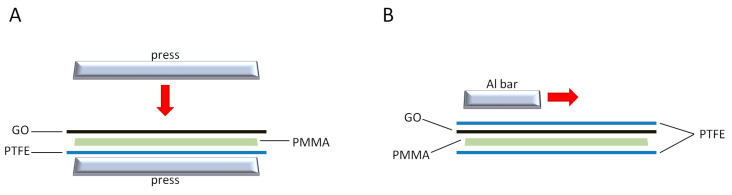
Schematic representation of a fabrication process of GO/PMMA_(P)_ (**A**) and GO/PMMA_(M)_ (**B**) composite materials.

For the fabrication of GO/PMMA composites, GO paper as well as SIMPLEX P (Stryker, Kalamazoo, MI, USA) radiopaque bone cement (prepared according to the manufacturer’s instructions with a full dose of liquid monomer) were applied. The monomer was mixed to the polymer manually under laboratory conditions. The PMMA cement was then inserted in a metal casting form and covered with GO paper. The bone cement was kept in place for 30 min to guarantee sufficient hardening. Screws were closed after 15 min. Two different methods were used to prepare combined samples including GO paper and bone cement (Scheme 1). 

In the first method, GO paper was placed on the bottom of a steel flat press (covered with poly(tetrafluoroethylene), PTFE, sheet to simplify cement detachment). The cement was spread on the upper PTFE sheet and then placed in contact with the GO paper. Then, the press was closed after 15 min to reach minimum thickness until the cement was polymerized (30 min). The samples prepared in this way were designated as GO/PMMA_(P)_. In the second method, a compound material was produced manually: GO paper was laid down on a PTFE sheet, and then the cement was spread on the upper PTFE sheet and then placed in contact with the GO paper. An aluminum bar was used to spread the cement on GO paper within the two PTFE sheets. In this case, spreading the cement was more difficult, and led to the formation of a non-homogeneous PMMA layer with GO paper broken up into little pieces, which may have been due to the shrinking and expanding behavior of PMMA during the cement hardening phase. The samples prepared in this way were designated as GO/PMMA_(M)_. 

Consequently, four types of samples were analyzed: GO_(A)_/PMMA_(P)_, GO_(B)_/PMMA_(P)_, GO_(A)_/PMMA_(M)_, and GO_(B)_/PMMA_(M)_, all with PMMA as the surface layer. For the scanning electron microscopic (SEM) investigations, a FEI Quanta 250 field emission gun (Thermo Fisher Scientific, Hillsboro, OR, USA) was used under high vacuum conditions and 20 kV high tension. The micrographs were recorded with the Everhart–Thornley–Detector in secondary electron (SE) mode. The surfaces were sputter coated with a 10 nm thin layer of gold in order to provide sufficient electrical conductivity.

### 2.2. Tissue Harvest and Cell Culture 

Explant hMSPCs were established from tissue samples of spongiosa bone harvested during routine hip joint surgeries. The study protocol was approved by the local ethics committee (reference number 29-156ex16/17), and informed consent was obtained from each orthopedic surgery patient. The study included a total of three patients, aged between 25 and 35, excluding pregnant women and those suffering from local inflammatory processes, metabolic bone diseases, and impaired blood coagulation. The length of harvested bone samples was kept between 4 and 6 mm, and showed either cortical or cortical and cancellous structure. The samples were extensively rinsed with a phosphate-buffered saline (PBS; PAA Laboratory, Pasching, Austria) and transferred into 75 cm^2^ culture flasks (TPP, Trasadingen, Switzerland) with an appropriate volume of culture medium. For cell isolation and expansion, the samples were incubated in a humidified atmosphere (5% CO_2_, 37 °C).

### 2.3. Flow Cytometry

For a flow cytometric analysis, a total of 1 × 10^5^ hMSPCs were resuspended in 200 µL PBS. The characterization of cells was achieved with the use of commercial monoclonal antibodies, namely CD73 PE, CD90 APC, CD105 PE, CD45 APC-Cy7, CD34 APC, CD14 FITC, CD19 APC, and HLA-DR APC (BD Bioscience, San Jose, CA, USA). Titration had previously been used to determine the optimal amount of each antibody. Subsequently, two-color staining panels were used to present a combination of antibodies with non overlapping spectra. Negative cell lines and matched fluorochrome-conjugated isotype controls were applied to perform a background staining for antibodies. FACS LSR II System (BD Bioscience), FACSDiva software (BD Bioscience), and FCS Express software (De Novo Software, Los Angeles, CA, USA) were employed to perform a flow cytometry analysis, to acquire and to analyze obtained data, respectively. Rainbow Beads (BD Bioscience) was used to check the day-to-day consistency of measurements. To exclude debris and cell aggregates, viable cells were gated on forward scatter (FSC) and side scatter (SSC). hMSPCs were defined by their phenotype and analyzed on a logarithmic scale. Data from all donors were analyzed by collecting 10,000 events under identical parameters.

### 2.4. Multilineage Differentiation Analysis

A seeding density of hMSPCs was established at 10^4^ cells/cm^2^, and the cells were seeded in an expansion medium composed of Dulbecco’s modified Eagle’s medium (DMEM-F12; GIBCO Invitrogen), 10% FBS (Lonza, Braine-l’Alleud, Belgium), 1% L-glutamine, 1% penicilline/streptomycine, and 0.1% amphotericine B. Additionally, 100 nM dexamethasone, 0.1 mM ascorbic acid-2-phosphate, and 10 mM β-glycerophosphate (all Sigma Aldrich, St. Louis, MO, USA) were added to the differentiation medium to induce osteogenesis. Histochemical staining (Alkaline Phosphatase kit No. 85; Sigma Aldrich) was used to assay alkaline phosphatase (ALP) activity after 7 and 14 days of culture. According to the instructions of the manufacturer, ALP enzyme activity was calculated basing on the absorbance of p-nitrophenol phosphate (405 nm) [26]. Adipogenic differentiation was performed in a medium containing 100 nM dexamethasone, 50 µM indomethacine (Sigma Aldrich), and 0.135 IE/mL insulin (Novo Nordisk, Bagsværd, Denmark), and was detected by Oil Red O staining of the adipocyte specific fat vacuoles after 21 days of culture. Chondrogenic differentiation was initiated by culturing cells in DMEM-F12 supplemented with 10% FBS, 100 µM L-ascorbic acid, and 1 ng/mL TGF-β3 (Lonza). Alcian blue staining was applied to verify the production of glycosaminoglycans and mucopolysaccharides after 21 days of culture. Cells were then fixed with 10% formaldehyde and stained with 1% Alcian blue in 3% acetic acid solution at pH 2.5.

### 2.5. Real-Time RT-PCR

RNeasy Mini Kit and DNase-I treatment (Qiagen, Hilden, Germany) were used to isolate total RNA from undifferentiated and osteogenic differentiated hMSPCs cultured on different GO surface modifications (on the GO-uppermost surface of the composites) on day 21. A total of 1 μg of RNA was reverse transcribed with iScriptcDNA Synthesis Kit, (BioRad Laboratories Inc., Hercules, CA, USA) using a blend of oligo(dT) and random hexamer primers. SsoAdvanced Universal SYBR Green Supermix (Bio-Rad) and CFX96 Touch (BioRad) were used for the amplification and measurements, respectively. A standard 3-step PCR temperature protocol (annealing temperature of 60 °C) was used for each qPCR, and was followed by a melting curve protocol both to confirm a single gene-specific peak and to detect primer dimerization. ΔΔCt method was used for the relative quantification of expression levels, and was based on the geometric mean of the internal controls TBP (TATA-box binding protein), RPLP0 (ribosomal protein, lateral stalk, subunit P0), and B2M (β-2 microglobulin), respectively. The expression levels (Ct) of the target genes were normalized to the reference genes (ΔCt), and the difference between the ΔCt value of the test sample and the ΔCt of the control sample gave the ΔΔCt value. Consequently, the expression ratio was calculated as 2^ΔΔCt^. Three QuantiTect primer assays (Qiagen) were selected for real time RT-PCR, namely ALPL, SPARC, and BMP2.

### 2.6. Statistical Analysis 

Differences between groups were evaluated by means of a Student’s unpaired *t*-test and the exact Wilcoxon test with the PASW statistics 18 software (IBM Corporation, Somers, NY, USA). Two-sided *p*-values (*p* < 0.001 ***; *p* < 0.01 **; *p* < 0.05 *) were considered statistically significant. SigmaPlot^®^ 14.0 (Systat Software Inc., San Jose, CA, USA) was used to make graphical representations.

## 3. Results

### 3.1. Surface Characterization of GO/PMMA 

**Figure 1:**
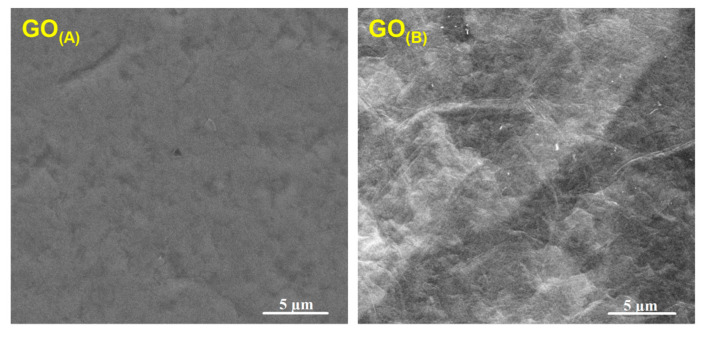
SEM micrographs presenting surface morphology of GO_(A)_ and GO_(B)_ paper.

The protocol of GO paper fabrication resulted in the formation of a self-supporting, uniform, and black material, with the average thickness of 5 ± 1 µm for GO_(A)_ and 16 ± 1 µm for GO_(B)_, and the average specific weight of 0.87 ± 0.08 mg/cm^2^ for GO_(A)_ and 2.90 ± 0.08 mg/cm^2^ for GO_(B)_. The morphology of GO_(A)_ and GO_(B)_, as presented in the SEM images (Figure 1), showed some wrinkles on the surfaces which were the most probably the edges of graphene oxide, revealing strong adhesion between GO platelets. Overall, the surface of GO paper was relatively smooth, and there were no obvious defects (pores or cracks) observed. 

**Figure 2:**
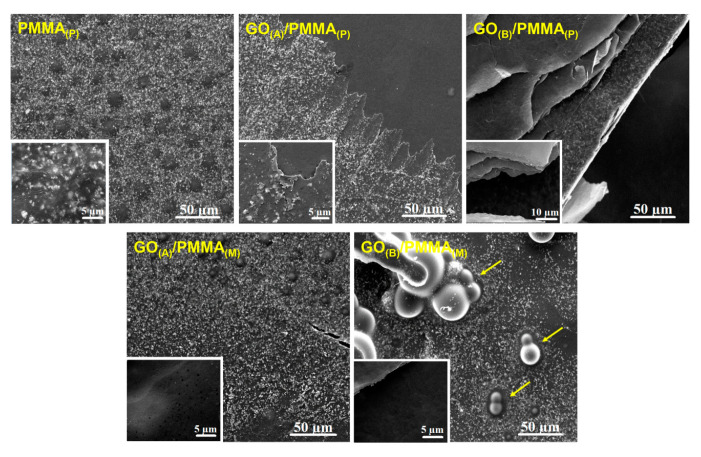
SEM micrographs presenting surface morphology of GO_(A)_/PMMA_(P)_, GO_(B)_/PMMA_(P)_, GO_(A)_/PMMA_(M)_, and GO_(B)_/PMMA_(M)_ (arrows indicate discussed surface structures), as well as untreated PMMA, with high magnification images as the insets.

As demonstrated in SEM micrographs of GO/PMMA composites (Figure 2), PMMA was covering the surface of GO paper, and more uniform surface was obtained when a thin GO_(A)_ paper was used as a filler. The interface between GO and PMMA can be seen as a border region, particularly in the SEM image of GO_(A)_/PMMA_(P)._ GO_(A)_ paper was thickly coated with PMMA, while the presence of a thick GO_(B)_ paper was found to introduce wrinkles to the surface of polymer composite. Moreover, GO_(B)_ paper seemed to protrude cleanly from the fracture site. The surface of GO_(B)_/PMMA_(M)_ was observed to exhibit a significant number of voids and pores.

### 3.2. hMSPC Characterization and Multilineage Differentiation Analysis

**Figure 3:**
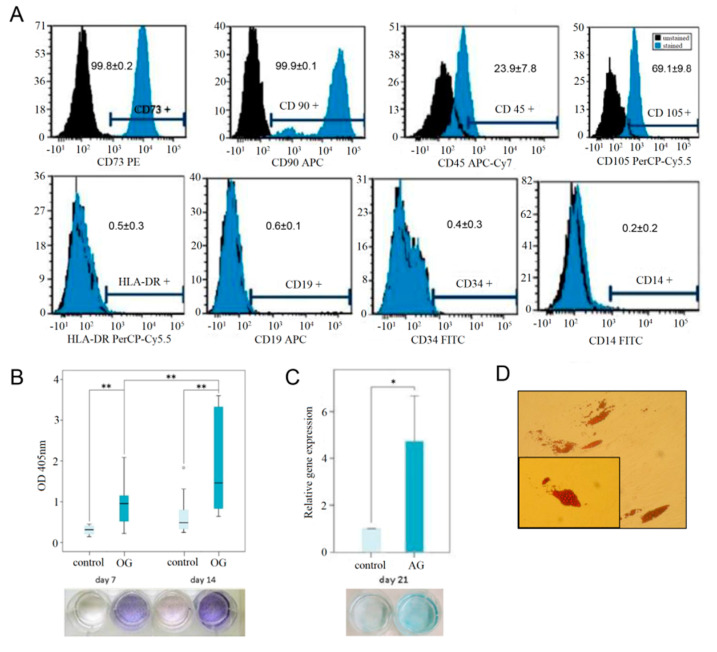
hMSPC characterization and multilineage differentiation analysis. The used hMSPCs were characterized according (**A**) the positive expression of CD73, CD90, CD105, and negative expression CD14, CD19, CD34, CD45, and HLA-DR using multicolor fluorescence-activated cell sorting analyses. The values indicated the percentage of positively stained cells. The capacity for multilineage differentiation potential was confirmed by (**B**) ALP staining for osteogenic differentiation, (**C**) Alcian blue staining and the expression of aggrecan for the chondrogenic differentiation, and (**D**) the Oil Red O staining of lipid droplets for the adipogenic lineage; *p* < 0.01 **; *p* < 0.05 *).

Cells providing morphologic characteristics of human primary MSPCs (mononuclear, fibroblast-like, spindle shaped, plastic-adherent) were isolated from all samples within 4–8 days. hMSPCs showed a positive expression of CD73 (99.8 ± 0.1%), CD90 (99.9 ± 0.1%), CD105 (69.1 ± 9.8%) of gated cells. The typical forward/side scatter characteristics of 71.5 ± 4.9% were gated. The negativity for CD14 (0.2 ± 0.2%), CD19 (0.6 ± 0.1%), CD34 (0.4 ± 0.3%), CD45 (23.9 ± 7.8%), and HLA-DR (0.5 ± 0.3%) confirmed the phenotype of MSPCs (Figure 3A). 

ALP activity was measured of absorbance (optical dense, OD) of p-nitrophenol in supernatant at the wavelength of 405 nm over 14 days (Figure 3B). ALP expression was detected on day 7 and day 14, respectively, when the cells were osteogenically differentiated with a significant increase (*p* < 0.001). No expression of ALP was observed in any of the samples of undifferentiated negative controls. Due to the interaction of the cationic dye Alcian blue and acid glycosaminoglycans, augmented blue coloration was noticed for chondrogenic differentiated hMSPCs, and not for undifferentiated controls (Figure 3C). As a result of chondrogenic differentiation, a 4.7-fold increase (*p* < 0.05) was noticed for the expression of aggrecan. In order to demonstrate the multilineage ability, hMSPCs were also differentiated in the adipogenic lineage. The adipogenic cell differentiation was demonstrated with the formation of lipid vacuoles which were visualized by Oil Red O staining on day 21 (Figure 3D). These results explicitly characterized our primary cells as hMSCPs.

### 3.3. Efficiency of Osteogenic Differentiation

**Figure 4:**
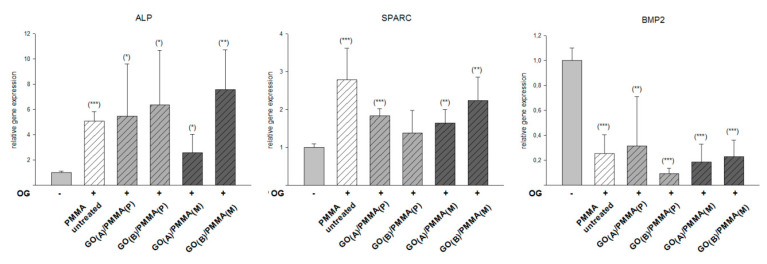
The expression of specific osteogenic markers (ALP, SPARC, and BMP-2) of hMSPCs cultured in the presence of osteogenic differentiation medium for 21 days on the surface of GO/PMMA composites as well as untreated PMMA, compared with undifferentiated hMSPCs cultivated in expansion medium as the negative control; *p* < 0.001 ***; *p* < 0.01 **; *p* < 0.05 *.

ALP, SPARC, and BMP-2 assays were performed to assess the mineralization of hMSPCs cultured on the surface of GO_(A)_/PMMA_(P)_, GO_(B)_/PMMA_(P)_, GO_(A)_/PMMA_(M)_ and GO_(B)_/PMMA_(M)_, as well as untreated PMMA. Consequently, the results shown in Figure 4 describe how strongly the expression of individual markers was increased by the osteogenic differentiation medium, with the undifferentiated hMSPCs as the control. As demonstrated by ALP assay, all investigated surfaces led to a significant increase in mineral deposition, with the most pronounced effect noticed for GO_(B)_/PMMA_(M)_ (8-fold increase when compared with a control). The same material was also found to lead to the significant increase in the expression of SPARC (2-fold increase with respect to control), even though the highest relative gene expression (3-fold increase with respect to control) was noticed for unmodified PMMA. All investigated surfaces, including GO/PMMA composites as well as unmodified PMMA samples, were shown to decrease the relative BMP-2 expression from 7 to 9 times when compared with a control. 

**Figure 5:**
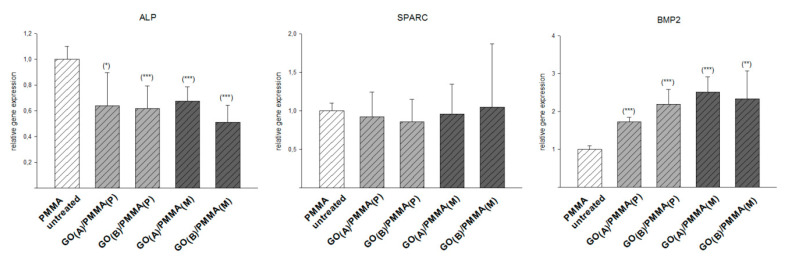
The expression of specific osteogenic markers (ALP, SPARC, and BMP-2) of hMSPCs cultured in the absence of a specific induction medium for 21 days on the surface of GO/PMMA composites as well as untreated PMMA; *p* < 0.001 ***; *p* < 0.01 **; *p* < 0.05 *.

The relative gene expression profiles of hMSPCs cultured in normal expansion medium were analyzed with respect to ALP, SPARC and BMP-2, and compared with an unmodified PMMA as the control. Consequently, the results presented in Figure 5 describe the expression of the individual markers by the osteogenic differentiation medium in relation to an unmodified PMMA control These results showed the unchanged SPARC expression, simultaneously with the decrease in ALP expression (approximately 2-fold) and a significant increase of BMP-2 expression (from 2-fold for GO_(A)_/PMMA_(P)_ to 2.5-fold for GO_(A)_/PMMA_(M)_) for GO/PMMA composites with respect to a PMMA control. 

**Figure 6:**
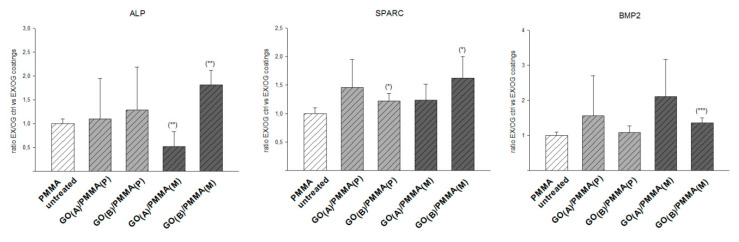
The expression of specific osteogenic markers (ALP, SPARC, and BMP-2) of hMSPCs cultured for 21 days on the surface of GO/PMMA composites with respect to a PMMA control; *p* < 0.001 ***; *p* < 0.01 **; *p* < 0.05 *.

To estimate the efficiency of osteogenic differentiation, GO/PMMA coatings were compared with an unmodified PMMA as a control (Figure 6). This kind of evaluation was chosen to show the efficiency of the osteogenic differentiation ability of each group in relation to the PMMA control. Particularly, GO_(B)_/PMMA_(M)_ composite was found to improve the expression of osteogenic differentiation markers (2-fold increase for ALP, 1.5-fold increase for SPARC, and 2-fold increase for BMP-2), while GO_(A)_/PMMA_(M)_ was found to decrease ALP expression (2-fold). All other effects were assessed as not significant. 

## 4. Discussion

The greatest challenge of modern nanotechnology is to expand its scope from nanoscale into macroscale. Therefore, the possibility of fabricating free-standing, paper-like materials basing on nanoscale components is a subject of intensive research [27]. The protocol of GO paper fabrication, introduced by us, allowed formation of self-supporting, uniform films composed of stacked platelets of graphene oxide. The irregularity of surface of GO paper seemed to increase with its thickness, which is of special importance for further biological studies since higher roughness promotes cell adhesion [28].

GO paper is known to possess many functional groups, and could easily form hydrogen bonds with hydrophilic polymers. The presence of hydrophobic methacrylate groups in PMMA, however, discourages these interactions to occur. Therefore, since PMMA chains would rather remain in a coiled conformation, they are supposed to fit well into wavy structures of the nanosheets of GO paper leading to more efficient packing within intersheet gallery [29]. Consequently, SEM micrographs demonstrated a good interlocking between GO paper and PMMA matrix. Particularly GO_(A)_ paper was found to be thickly coated with PMMA, suggesting strong adhesion between this polymer and GO. GO_(B)_ paper, on the other hand, seemed to protrude from the fracture site, suggesting a weak interfacial bonding between GO_(B)_ and PMMA. This is consistent with some previous studies indicating that small sizes of GO sheets promote the formation of homogeneous composites [30]. Moreover, the presence of voids and pores on the surface of GO_(B)_/PMMA_(M)_ could be associated with the presence of unreacted residual monomer, which is volatile and is supposed to be released after polymerization [31]. This would mean that some double bonds present in GO paper could be attacked by the radical species formed during MMA polymerization, retarding or inhibiting the reaction of polymerization [24]. 

As a biological model, cells providing morphologic characteristics of human primary MSPCs (mononuclear, fibroblast-like, spindle shaped, plastic-adherent) were isolated and cultured on the surface of GO/PMMA composites. The phenotype of hMSPCs was confirmed according to the criteria of the International Society for Cellular Therapy [32] for defining multipotent mesenchymal stromal cells. In addition, hMSPCs were successfully differentiated towards the osteogenic, chondrogenic, and adipogenic lineage, which was confirmed by ALP expression, Alcian blue staining, aggrecan expression, and Oil Red O staining. To assess the efficiency of osteogenic differentiation of hMSPCs cultured onto PMMA as well as GO/PMMA composites, three differentiation markers were analyzed, namely alkaline phosphatase (ALP), secreted protein acidic and rich in cysteine (SPARC), and bone morphogenetic protein-2 (BMP-2). ALP is a metalloenzyme playing an important role in the mineralization of tissue cells [33]. ALP is found to act as both a mineralization promoter by increasing the local concentration of phosphate, as well as an inhibitor of mineral formation by decreasing the concentration of extracellular pyrophosphate. Since ALP is observed to be highly expressed in mineralized cells, it can be used to predict their bone forming capacity under different conditions. Osteonectin (SPARC), on the other hand, is the most abundant non-collagenous extracellular matrix protein present in bone [34]. SPARC gene dosage has a dramatic effect on bone volume and is one of critical regulators of bone remodeling, calcium turnover and an initiator of mineralization [34,35]. Therefore, SPARC can be used for the examination of osteogenic differentiation. Another marker for osteogenic differentiation is bone morphogenetic protein-2 (BMP-2), which is known as a potent osteogenic factor with roles in both normal bone healing and pathological bone formation in soft tissues [36]. BMP-2 is found to facilitate osteogenic differentiation through inducing ALP activity, promoting mineralization and enhancing adherence of cells [37].

As demonstrated by ALP assay, all investigated surfaces led to a significant increase in mineral deposition. This effect was accompanied with a decrease in BMP-2 expression, which might suggest that the presence of a differentiation medium had a stronger effect on the osteogenic differentiation than BMP-2, resulting in down regulation of the latter. Nevertheless, the expression of specific osteogenic markers, such as ALP and SPARC, was found to be significantly increased by the osteogenic differentiation medium. In the absence of a specific induction medium, on the other hand, the cells cultured on GO/PMMA composites were found to be able to induce an increase in BMP-2. In this way, GO/PMMA composites were shown to be able to drive cellular differentiation without any addition of osteogenic supplements, just as reported for hMSCs in collagen matrices subjected cyclic tensile strain [38], even though the process of osteogenic differentiation was found to be much more effective when cells were culture in the presence of a medium trigger. 

The comparison of GO/PMMA coatings with an unmodified PMMA as a control suggested that among all investigated GO/PMMA composite materials, GO_(B)_/PMMA_(M)_ composite was the most efficient inducer of osteogenic differentiation, particularly basing on the relative expression of ALP (other changes were found to be statistically non-significant). This effect should be assigned to its surface morphology, as exhibiting a significant number of voids and pores. As presented by Abagnale et al. [39], specific patterns present on the surface can boost differentiation of MSCs towards specific cell types. These patterns should be in the micrometer range to be able to support the differentiation processes initiated by induction media, and this requirement is met particularly by GO_(B)_ paper and GO_(B)_/PMMA_(M)_ composite. All these data suggest that GO/PMMA composites, particularly GO_(B)_/PMMA_(M)_ produced manually with a thick GO paper, may direct hMSPCs toward osteogenic differentiation and can serve as promising materials in bone tissue engineering. The properties of GO/PMMA make this material advantageous for potential applications as screws or implants fixation. Previous studies [25,40] showed that PMMA filled with GO conforms to physicochemical and mechanical demands of these clinical applications. In this study, we have shown that incorporation of GO into PMMA matrix may provide an additional functionality to the resulting composite material, enhancing its biocompatibility through facilitating osteogenesis. Still, before the introduced material could be considered as a bone cement, further studies should include a comprehensive biomechanical characterization of GO/PMMA.

## 5. Conclusions

In this paper, we present the potency of GO/PMMA composites to induce osteogenic differentiation of hMSPCs. Through the analysis of three differentiation markers, namely ALP, SPARC, and BMP-2 in the presence and in the absence of a specific induction medium, we were able to assess the efficiency of osteogenic differentiation of hMSPCs cultured on four types of GO/PMMA composites, differing in the thickness of GO paper as well as the method of fabrication of the composite. All investigated GO/PMMA composite materials were found to effectively induce osteogenic differentiation, and to outperform both unmodified PMMA and a negative control (undifferentiated hMSPCs). Among GO/PMMA composite materials, a composite produced manually with a thick GO paper (GO_(B)_/PMMA_(M)_) acted as the most efficient inducer of osteogenic differentiation, particularly basing on the relative expression of ALP (other changes were found to be statistically non-significant). Since GO_(B)_/PMMA_(M)_ was the composite surface possessing a significant number of voids and pores, its developed surface morphology was supposed to be responsible for directing hMSPCs toward osteogenic differentiation. In this way, GO/PMMA composites, and particularly GO_(B)_/PMMA_(M)_, were shown as promising materials in bone tissue engineering.
